# Iliosacral Screw Fixation for Treating Sacral Fractures: A Modified Pedicle‐Guided Fixation Technique in Prone Position

**DOI:** 10.1111/os.70439

**Published:** 2026-09-21

**Authors:** Ben‐Mao Liu, Yu‐Hsin Liu, Ting‐Shuo Hsu, Chih‐Yu Chen, Chang‐Jung Chiang, Yueh‐Ying Hsieh

**Affiliations:** ^1^ Department of Orthopedics, Shuang Ho Hospital Taipei Medical University New Taipei City Taiwan; ^2^ Department of Biomedical Engineering, College of Medicine and College of Engineering National Taiwan University Taipei Taiwan; ^3^ Department of Orthopaedics, School of Medicine, College of Medicine Taipei Medical University Taipei Taiwan; ^4^ School of Biomedical Engineering Taipei Medical University New Taipei City Taiwan; ^5^ Department of Orthopedics, Hsin Kuo Min Hospital Taipei Medical University Taipei Taiwan

**Keywords:** iliosacral screw, iliosacral screw fixation, iliosacral screw placement, prone position, sacral fractures

## Abstract

**Background:**

Sacral fractures are posterior pelvic ring injuries which mechanisms range from high‐energy trauma to low‐energy falls in osteoporotic patients. In surgical treatment, percutaneous approaches have demonstrated significant shorter operation time and less soft tissue damage. In this study, we present a method of iliosacral (SI) screw insertion in prone position, with modified pedicle‐guided technique.

**Methods:**

The present study is a retrospective case series of 22 patients for SI screw fixation from February 2019 to September 2023 under our method. Surgical technique was described in detail with images. Perioperative clinical parameters were analyzed. Activity status, complications, data of 10‐point visual analogue scale (VAS) for pain and further functional outcomes using the Majeed Pelvic Score (MPS) were recorded. In addition, computed tomography (CT) was routinely performed at 6 months postsurgery to inspect the quality of screws position.

**Results:**

Excellence rate of screw position was 97% upon CT. Two patients had screws partially contacting the cortical bone but not violating sacral foramen, and no neurological symptoms were noted. All screws were secure from possible iatrogenic nerve injuries. VAS scores improved significantly over time and most patients recorded excellent functional recovery. One untoward complication occurred involving unexpected cement extravasation after sacroplasty. No associated neurologic compromise, or clinical evidence of venous emboli thrombosis was reported.

**Conclusion:**

Our method aims at reducing operation time and radiation exposure to surgeons as conventional two‐dimensional fluoroscopy was enough to identify safe insertion corridors in SI screw placement. Performing SI fixation in prone position is also advantageous when dealing with obese patients and concomitant spinal injuries.

## Graphical abstract

Modified pedicle‐guided fixation technique for iliosacral screw placement in prone position reduces operation time and radiation exposure as conventional two‐dimensional fluoroscopy was enough to identify safe insertion corridors. Prone position is also advantageous when dealing with obese patients and concomitant spinal injuries.

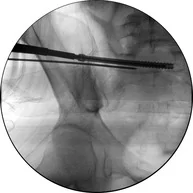

**Abbreviations**
AP: anteroposterior
CT: computed tomography
K‐wire: Kirschner wire
MRI: magnetic resonance imaging
SI: iliosacral
VAS: visual analogue scale

## 1 Introduction

Sacral fractures are posterior pelvic ring injuries which mechanisms range from high‐energy trauma to low‐energy falls in osteoporotic patients. Most fractures cause pelvic ring instability which disrupts spinopelvic balance and overall biomechanics necessitating surgical treatment. If left untreated, the instability often leads to chronic pain, with risks of neurological complications including severe radiculopathy, persistent motor weakness or even more devastating loss of bowel and bladder function. Suboptimal treatment also contributes to poor patient‐reported results and decline of quality of life. Purpose of fixation in traumatized patients is to restore pelvic structural stability and spinopelvic balance, increase mobility and reduce risks of subsequent complications due to prolonged immobilization [1]. While insufficiency fractures occur commonly as fragility fractures in elderly and present with occult low back pain, conservative treatments are generally unsuccessful [2]. Iliosacral (SI) screw fixation with or without sacroplasty has emerged as a safe and effective alternative to provide early symptomatic relief [3].

To date, minimally invasive techniques have become the mainstream in treating posterior pelvic ring injuries. Percutaneous approaches have demonstrated significant shorter operation time and less soft tissue damage when compared with traditional open fixation [4, 5]. Many reliable methods have been introduced for sacropelvic fixation, including percutaneous SI, transsacral, or sacral‐alar‐iliac screws. However, current percutaneous techniques remain technically demanding. They rely heavily on obtaining precise multiplanar fluoroscopic views to ensure safe osseous corridors, leading to considerable radiation exposure and prolonged setup times. In this study, we present a method of SI screw insertion in prone position, with modified pedicle‐guided technique.

Indications for SI screws include mostly sacral sagittal fractures, sacroiliac (SI) joint dislocations, and sacral insufficiency fractures [6]. Traditional supine approach in SI screw insertion utilizes intraoperative inlet and outlet views to establish the complex three‐dimensional anatomical structure of pelvis. However, the opacity of fluoroscopy can easily be affected by patients' factors such as sacral dysmorphism, obesity and intestinal gas buildup [7]. Frequent fluoroscopic imaging to prevent potential complications can also result in high radiation exposure. Placing SI screws in prone position, on the contrary, eliminates most of the above negative factors. Ideally fewer images are sufficient enough to provide safe working angles in screw placement. Iatrogenic complication such as vascular and neurological injury can be reduced. In addition, prone position is advantageous to the surgeon in simultaneously managing accompanied spinal or spinopelvic injuries without the need for risky intraoperative repositioning, thereby reducing total anesthesia time and surgical stress. Prone positioning also allows for a more direct anatomical reduction of posterior ring disruptions. Aim of the current study is to evaluate the technical feasibility, screw‐placement accuracy, and preliminary clinical outcomes of the modified prone‐position SI screw fixation in selected patients with sacral fractures.

## 2 Materials and Methods

### 2.1 Patients

The present study is a retrospective case series of 22 consecutive patients presented to our hospital for SI screw fixation between February 2019 to September 2023. The inclusion criteria were: (i) sacral fractures indicated for operative fixation; (ii) surgical intervention with the modified pedicle‐guided technique in the prone position; and (iii) confirmed diagnosis by computed tomography (CT) or magnetic resonance imaging (MRI). The exclusion criteria were: (i) presence of major neurological deficits; (ii) suspected pathological fractures; and (iii) prior spinopelvic surgery. All patients, including those diagnosed on MRI, underwent preoperative CT assessment for the presence of sacral dysmorphism. Multiplanar CT reconstructions were used to determine the osseous safe corridor and optimal screw trajectories. These images also allowed us to measure appropriate screw diameter and length. All subjects gave their written informed consents to participate in the study. Institutional Review Board approval was sought and obtained for this case series (IRB approval #TMU‐JIRB‐N202309054). Medical records and radiographic images of all eligible patients were examined retrospectively.

### 2.2 Surgical Technique

#### 2.2.1 Anestheisa and Positioning

Following the induction of general anesthesia, the patient is positioned prone on a radiolucent spine table in a knee–chest position with the abdomen hanging free. All pressure points are padded well. Fluoroscopic views should be checked before surgery that clear anteroposterior (AP), lateral, pelvic inlet and outlet views could be acquired.

#### 2.2.2 Fluoroscopic Setup

The gantry was first adjusted to a similar outlet view paralleling sacrum. Minor tilts were then made ensuring the transverse ridges of the sacral vertebra of interest overlapped in a line and bilateral pedicle visible in similar widths. At S1, it was adjusted to parallel the base of sacrum which gave a true sacral AP view.

*Precautions*: Securing this precise true AP view is critical to define safe osseous corridor.

### 2.3 Approach and Entry Point Localization

A 2.5‐mm Kirschner wire (K‐wire) as guide wire was used to project the trajectory and locate the entry point. The screw trajectory with the planned distance from skin incision can be approximately measured on preoperative magnetic resonance images. A paramedian stab wound on back was made and the K‐wire was introduced until its tip made contact with bone. Iliac wing around the level of posterior gluteal line was reached at this point.

### 2.4 Pedicle‐Guided K‐Wire Advancement

Lateral fluoroscopy was obtained to confirm the level of interest and wire tip meeting the sacral dorsal surface. Light hammering followed by slow drilling was used to insert the K‐wire into sacrum through ilium passing through SI articulation. On AP view, The trajectory was made parallel to sacral base or transverse ridge to prevent too caudal or too cranial placement. The K‐wire was passed from lateral to medial margin of the pedicle. Right before penetrating the medial border, lateral fluoroscopy was used to guarantee the tip crossing the dorsal edge of sacral body. It ensured our trajectory not being too convergent that the K‐wire might enter into the sacral canal.

*Precautions:* Switching to lateral view immediately prior to penetrating the medial border is critical to avoid neurovascular injury.

### 2.5 Final Screw Insertion

**FIGURE 1:**
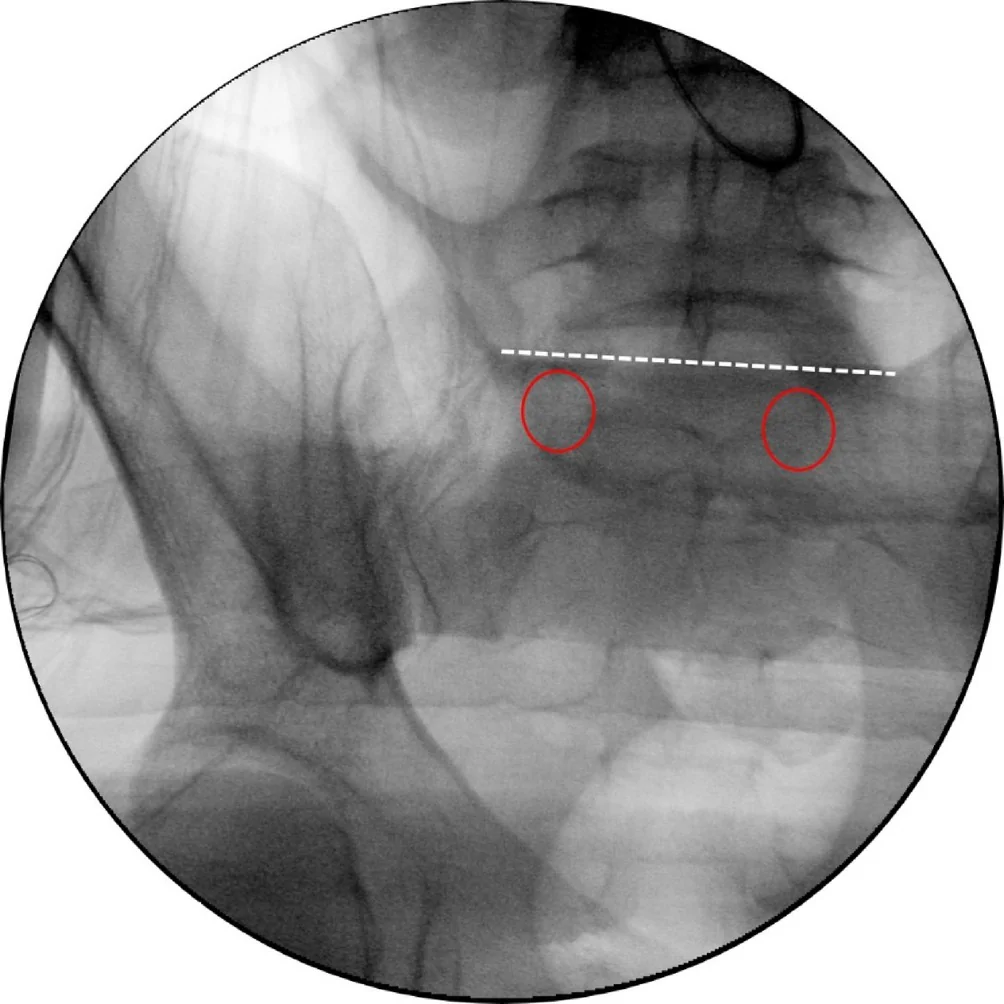
The initial fluoroscopic view demonstrates the base of sacrum overlapping in a single line (white dotted line), targeting the S1 pedicles with symmetric widths (red circles).

**FIGURE 2:**
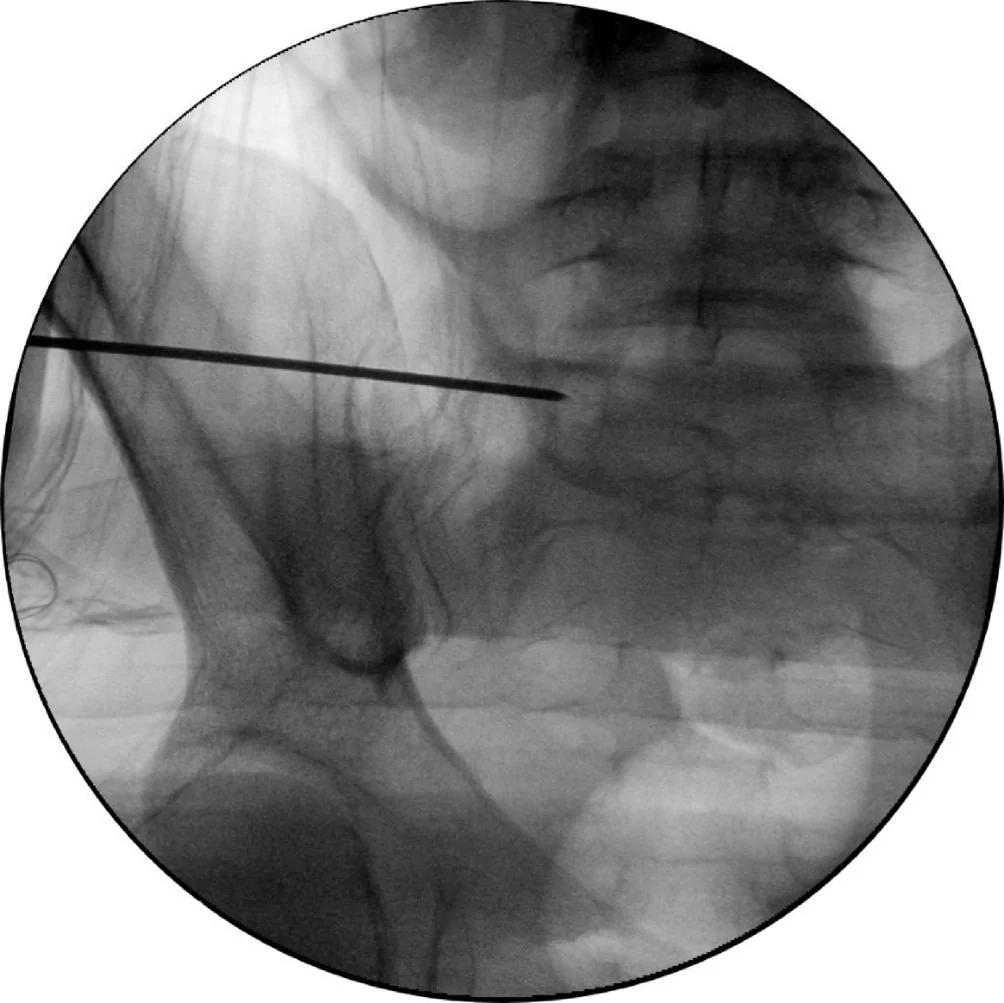
A 2.5‐mm Kirschner wire (K‐wire) is used to project the trajectory, aiming at the lateral entry point of the S1 pedicle, defined as the inferolateral margin at the base of the sacral superior articular process.

**FIGURE 3:**
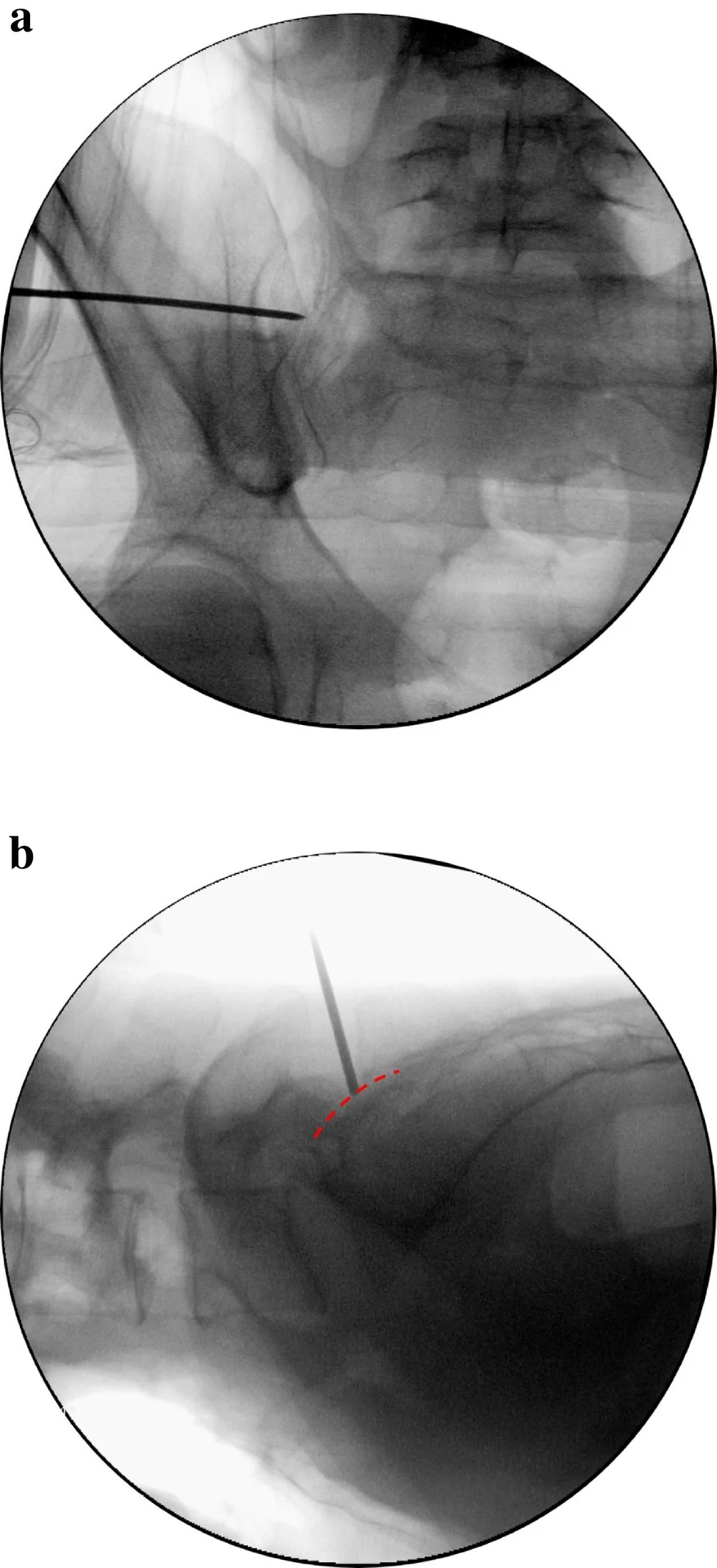
The K‐wire contacts the cortex of iliac wing prior to entering the SI joint. (a) In lateral view, the K‐wire tip ideally meets the sacral dorsal surface (red dotted line) (b).

**FIGURE 4:**
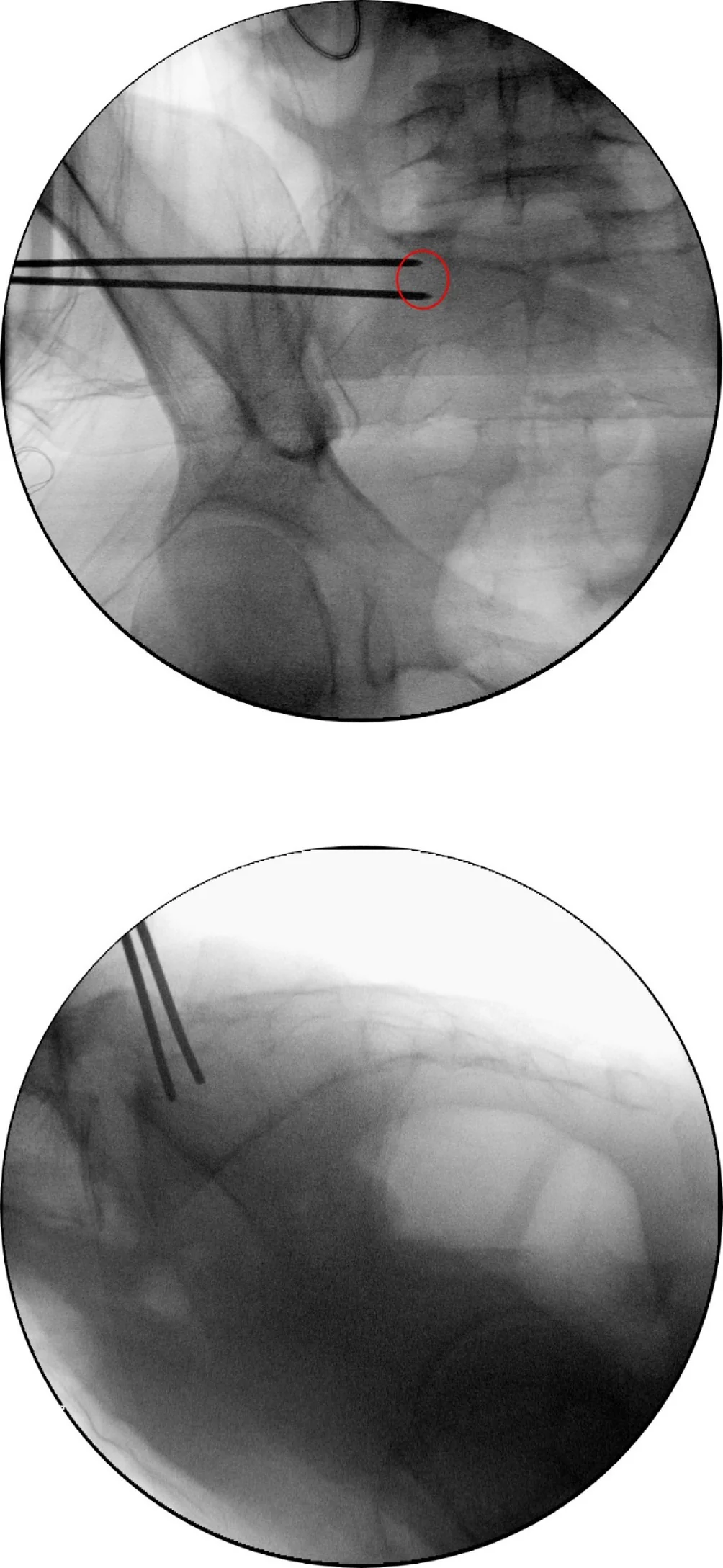
Two K‐wires are advanced into the left S1 pedicle (red circles), stopping immediately before penetrating the medial border. (a) The lateral view is checked to ensure the tips cross the dorsal edge of the sacral body (b).

**FIGURE 5:**
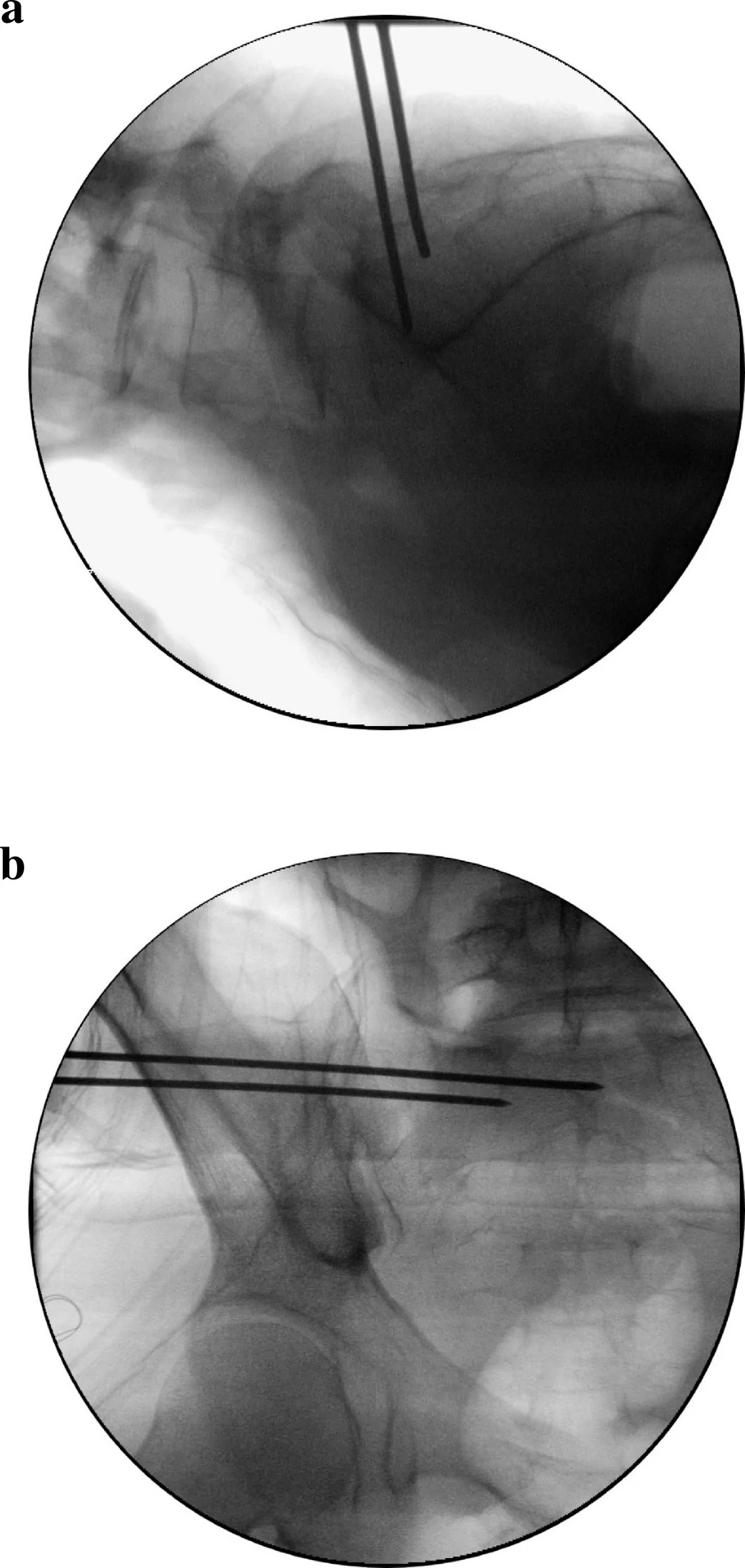
The K‐wires are continuously advanced toward the anterior cortex under lateral view. (a) The AP view shows that one of the K‐wires passes through the midline of the sacrum (b).

**FIGURE 6:**
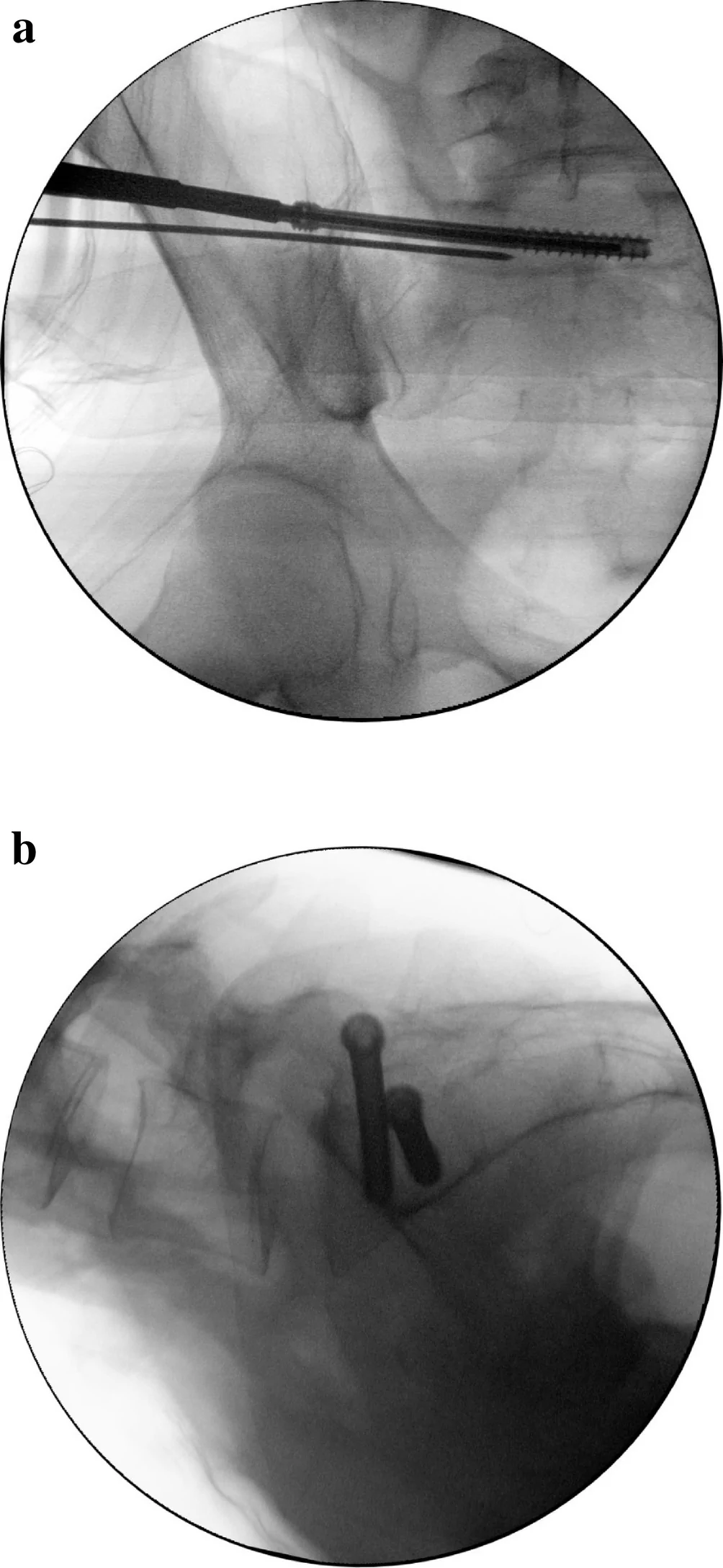
Following reaming, a screw with an appropriate length is inserted. (a) The final lateral view shows two SI screws inserted through S1 (b).

Once the track was secured, we can continue to purchase the wire under lateral view until it advanced enough and did not break the anterior cortex of sacral body. Reaming followed by screw insertion was done and K‐wire was removed. The procedure was summarized in Figures 1, 2, 3, 4, 5, 6.

### 2.6 Outcome Measurement

Perioperative clinical parameters including total operative time with average screw insertion time, intraoperative blood loss, and length of each stabbed wound incision were gathered. Fluoroscopic shots of each screw placement, including patient positioning and final documentary was analyzed. Standardized neurological examinations evaluating lower extremity motor and sensory functions as well as sphincter tone were performed preoperatively and postoperatively to monitor for sacral nerve root compromise. Routine clinical follow‐up in outpatient department was performed at 2, 4 weeks, 3, 6 months, and then on an ad hoc basis. Postoperative standard pelvic radiographic series (AP, lateral, inlet, and outlet views) were assessed. Activity status, complications, changes of the 10‐point visual analogue scale (VAS), and further functional outcomes using the Majeed Pelvic Score (MPS) [8] were recorded. In addition, CT was routinely performed in all patients at 6 months postsurgery to inspect the quality of screws position and condition of bone healing. Screw placement was assessed independently by two blinded reviewers, and interobserver reliability was calculated. We adopted Gras's criteria in assessing the excellence rate of screw placement by three categories as shown in Table 1 [9].

**TABLE 1:** The assessment criteria in screw placement.

Category	Description
Excellent	Completely within the cortex in cancellous bone
Good	Partially contacting the cortical bone (neural foramina or spinal canal)
Poor	Penetrating the cortical bone

### 2.7 Statistical Analysis

Statistical analyses were performed using SPSS 22.0 (IBM Corp., Armonk, NY, USA). Continuous data were expressed in terms of mean and standard error of the mean. Paired *t*‐test was used to compare the VAS scores between the preoperative baseline and all respective postoperative assessment periods. The Majeed Pelvic scoring system was adjusted based on preinjury working status allowing a maximum of 100 points for patients working and 80 points for those who were not. A *p*‐value < 0.05 was considered statistically significant. Interobserver reliability was calculated using Cohen's kappa coefficient (*κ*) to verify assessment consistency.

## 3 Results

### 3.1 Patient Characteristics

Of 22 patients, 17 sustained traumatic sacral fractures and 5 sustained insufficiency fractures. In all cases, screws were inserted in both S1 and S2 levels to achieve stable fixation under the strategy of a minimum of two unilateral screws for unilateral fractures and a minimum of four screws for bilateral fractures. Unilateral fixation was performed in 14 patients, and bilateral fixation in eight patients. Additionally, sacroplasty with polymethylmethacrylate (PMMA) cement was performed in 17 patients with confirmed insufficiency fractures and osteoporosis (*T* score < −2.5), who were further analyzed to assess cement injection. Twelve patients used high viscosity cement and five used low viscosity cement. All procedures were well tolerated and all patients (*n* = 22) were clinically followed‐up for a minimum of 6 months postsurgery. Median follow‐up time was 14.5 months (range 8–26 months). No patients were dropped from our study, with no missing data at any time point. The demographic data of patients was shown in Table 2. Perioperative clinical parameters with a separate sacroplasty subgroup were presented in Table 3. In addition, individual patient characteristics were summarized in Table 4.

**TABLE 2:** Patients' demographic data.

Variables	Patients (*n* = 22)
Age (mean, range)	70.6 years (25–92)
Gender	
Male	4 (18%)
Female	18 (82%)
Body mass index (mean, range)	22.4 kg/m^2^ (16.4–34.6)
Associated comorbidity	
Hypertension	11 (50%)
Diabetes	9 (41%)
Cardiac	7 (32%)
Working capacity	
Working	13 (59%)
Not working	9 (41%)
Injury mechanism	
High energy trauma	5 (23%)
Fall from stand	12 (55%)
No obvious trauma history	5 (23%)
Denis classification	
Zone I	4 (18%)
Zone II	18 (82%)
Associated spinal injuries	10 (45%)
Screw fixation	
Unilateral	14 (64%)
Bilateral	8 (36%)
Sacroplasty	17 (77%)

**TABLE 3:** Perioperative clinical parameters (mean ± standard deviation).

Parameters	
Total operative time (min)	51.4 ± 11.2
Time per screw placement (min)	14.9 ± 4.8
Fluoroscopic shots per screw placement	27.0 ± 9.7
Intraoperative blood loss (mL)	36.4 ± 9.2
Length per stabbed wound incision (cm)	1.65 ± 0.3
Sacroplasty (*n* = 17)	
Additional operative time (min)	9.7 ± 2.6
Additional fluoroscopic shots per screw	5.6 ± 1.6

**TABLE 4:** Individual characteristics of 22 patients treated with prone SI screw fixation.

Patient	Denis^a^	Screws	Sacroplasty	Fluoroscopic shots	Implants^b^	Screw grade	Complications	Fracture union	VAS trend^c^	Final walking status
1	II	4	Y	119	55/55/65/70 mm	All excellent	None	6 months	8,3,2,1,1	Independent
2	I	4	Y	105	65/65/70/70 mm	All excellent	None	6 months	7,4,1,1,1	Independent
3	II	2	N	54	80/85 mm	All excellent	None	6 months	6,3,0,0,0	Independent
4	II	3	Y	77	80/90/90 mm	All excellent	None	6 months	7,2,2,1,0	Independent
5	II	4	Y	118	65/65/75/80 mm	All excellent	None	6 months	8,4,1,1,1	Independent
6	II	4	Y	98	60/65/80/80 mm	All excellent	None	6 months	8,3,3,2,2	Partially dependent
7	I	2	Y	58	75/85 mm	All excellent	None	6 months	6,4,1,1,1	Independent
8	II	2	N	60	70/70 mm	1 excellent, 1 good	None	6 months	9,3,1,0,0	Independent
9	II	3	Y	80	75/75/80 mm	All excellent	None	6 months	7,3,2,2,0	independent
10	II	3	Y	82	80/85/90 mm	All excellent	None	6 months	7,3,2,1,1	Independent
11	II	4	Y	89	55/65/70/70 mm	All excellent	Cement extravasation	6 months	6,4,0,0,0	Independent
12	II	2	N	55	90/100 mm	All excellent	None	6 months	5,3,2,2,2	Independent
13	II	3	Y	83	80/95/95 mm	All excellent	None	6 months	8,2,1,1,1	Partially dependent
14	I	2	Y	59	95/105 mm	All excellent	None	6 months	8,3,0,0,0	Independent
15	II	2	Y	67	75/80 mm	All excellent	None	6 months	6,4,3,2,2	Independent
16	I	2	Y	60	100/105 mm	All excellent	None	6 months	7,4,1,1,1	Independent
17	II	4	Y	103	70/70/80/80 mm	3 excellent, 1 good	None	6 months	6,3,2,2,2	Independent
18	II	3	Y	87	80/80/100 mm	All excellent	None	6 months	8,3,1,1,0	Independent
19	II	2	N	49	75/80 mm	All excellent	None	6 months	9,3,3,2,2	Independent
20	II	3	N	67	55/55/65 mm	All excellent	None	6 months	9,3,2,1,0	Independent
21	II	4	Y	118	75/75/80/85 mm	All excellent	None	6 months	7,4,2,1,1	Partially dependent
22	II	4	Y	97	65/65/75/85 mm	All excellent	None	6 months	9,3,3,2,1	Independent

Abbreviations: N, no; Y, yes.

^a^ Zone classification.

^b^ All screws used for fixation were 6.5 mm headless compression screws (DePuy Synthes).

^c^ VAS trend presented in the order of preoperative, immediate‐, 1‐month‐, 3‐month‐ and 6‐month‐postoperative.

### 3.2 Follow‐Up and Radiographic Evaluation

**FIGURE 7:**
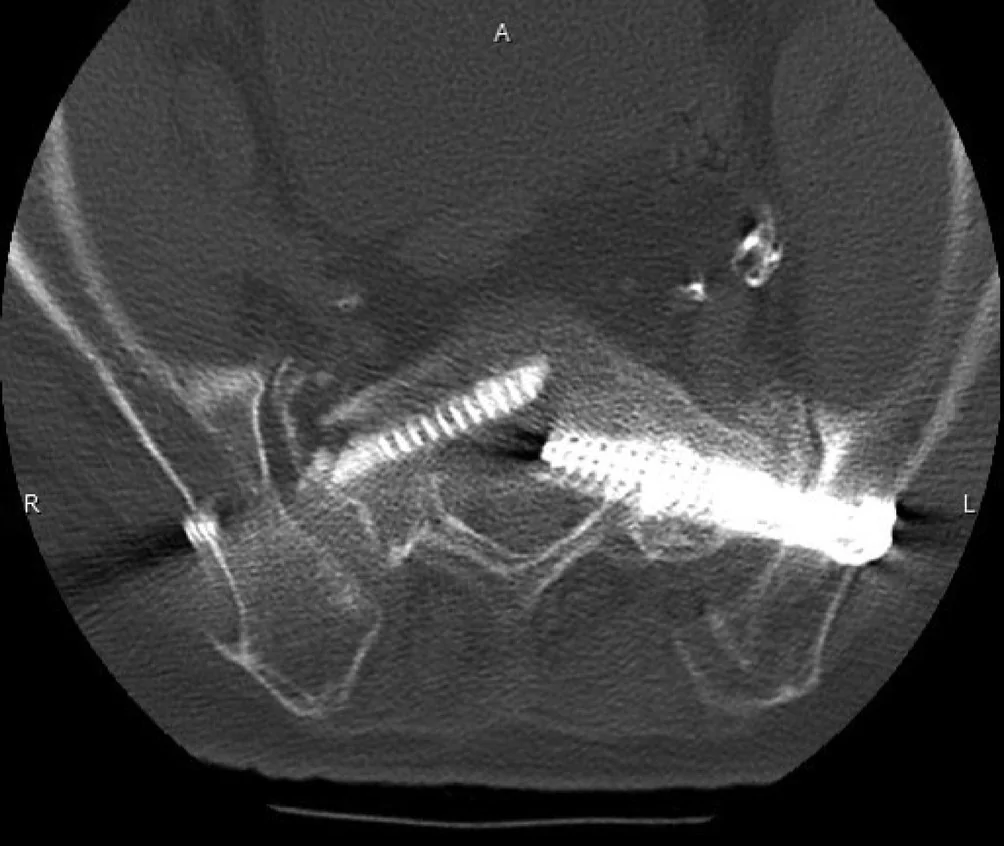
CT image showing the left SI screw partially contacts the cortical bone but does not violate the sacral foramen.

We arranged rehabilitation program for each patient. Every patient was able to roll over in bed and sit‐to‐stand with aid of walker independently before discharge. Each hospital stay was no more than 5 days. During follow‐up at 1 month postoperatively, all patients walked into our outpatient department with or without aid of walker. By 2 months postsurgery, all patients were able to return to previous walking activity status under full weight‐bearing. Postoperative pelvic plain films were taken every visit to assess fracture healing and screw placement. CT was performed at the sixth month and all patients showed definitive fracture union by the presence of continuous bridging bone across the fracture site. The quality of screw placement was assessed using the mentioned criteria. Of a total of 66 screws placed, no screw malposition was observed in intraoperative images or postoperative plain films, whereas in CT scans two patients had one screw each partially contacting the cortical bone but not violating sacral foramen (Figure 7). No neurological symptoms were seen in these patients and no revision surgery was necessary. There was no poor screw placement, indicating that all our cases were secure from iatrogenic nerve injuries. The excellence rate of screw position upon CT images was 97%. Interobserver reliability for CT image assessment was also excellent as the two independent reviewers agreed on all the grading of 66 total screws (*κ* = 1).

### 3.3 Clinical Outcomes and Functional Evaluation

**FIGURE 8:**
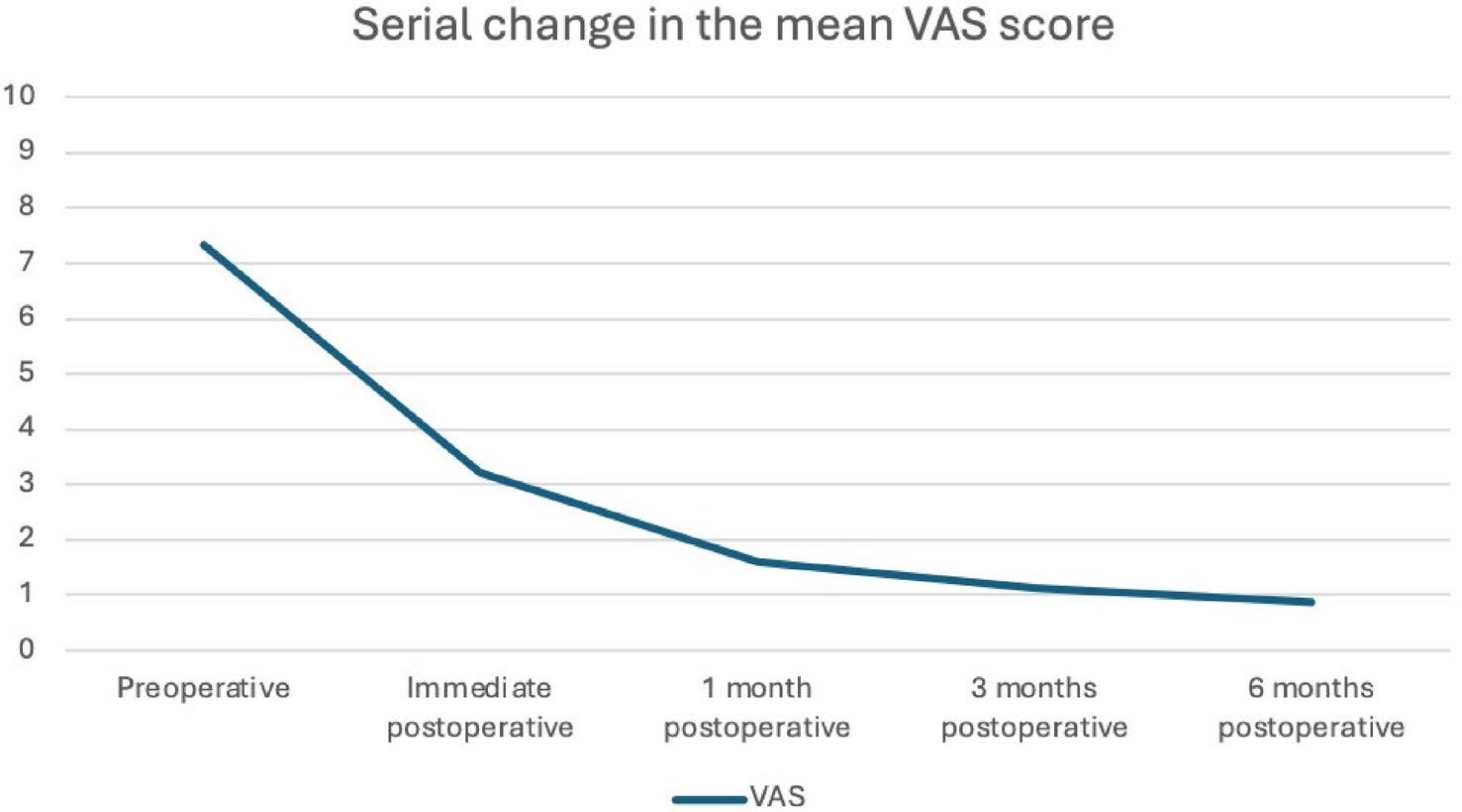
Change in the mean VAS score throughout the follow‐up period.

VAS of pain questionnaire was given preoperatively, immediate postoperatively, and at clinic returns. Preoperative mean VAS score of low back and buttock pain was 7.32 ± 1.14. Most patients have described a notable pain relief immediately postsurgery with mean score at 3.23 ± 0.60. After 1 month follow‐up, it decreased to 1.59 ± 0.94, and further drop to 1.14 ± 0.71 and 0.86 ± 0.77 at 3 and 6 months, respectively. The results demonstrated highly significant pain reduction across all assessment period compared with baseline (*p* < 0.001) and improvement of pain was the most significant from immediate to first month postoperatively (*p* < 0.001, Cohen's *d* = 4.21; 95% CI, 3.72–4.46). Serial change was shown in Figure 8. Activity status and functional outcomes were also evaluated using the MPS at 6‐month follow‐up. Mean MPS was 90.38 ± 3.18 and 73.56 ± 2.88 for patients working and not working respectively, indicating excellent functional recovery in most patients. Furthermore, assessment of functional walking status at final follow‐up revealed that 19 patients were able to ambulate independently. The remaining three patients who were osteoporotic elderly individuals, required crutches matching their pre‐injury status. None of our patients experienced wound poor healing or infections, and there was no broken screw or implant loosening seen among our postoperative CT scans.

### 3.4 Complications

**FIGURE 9:**
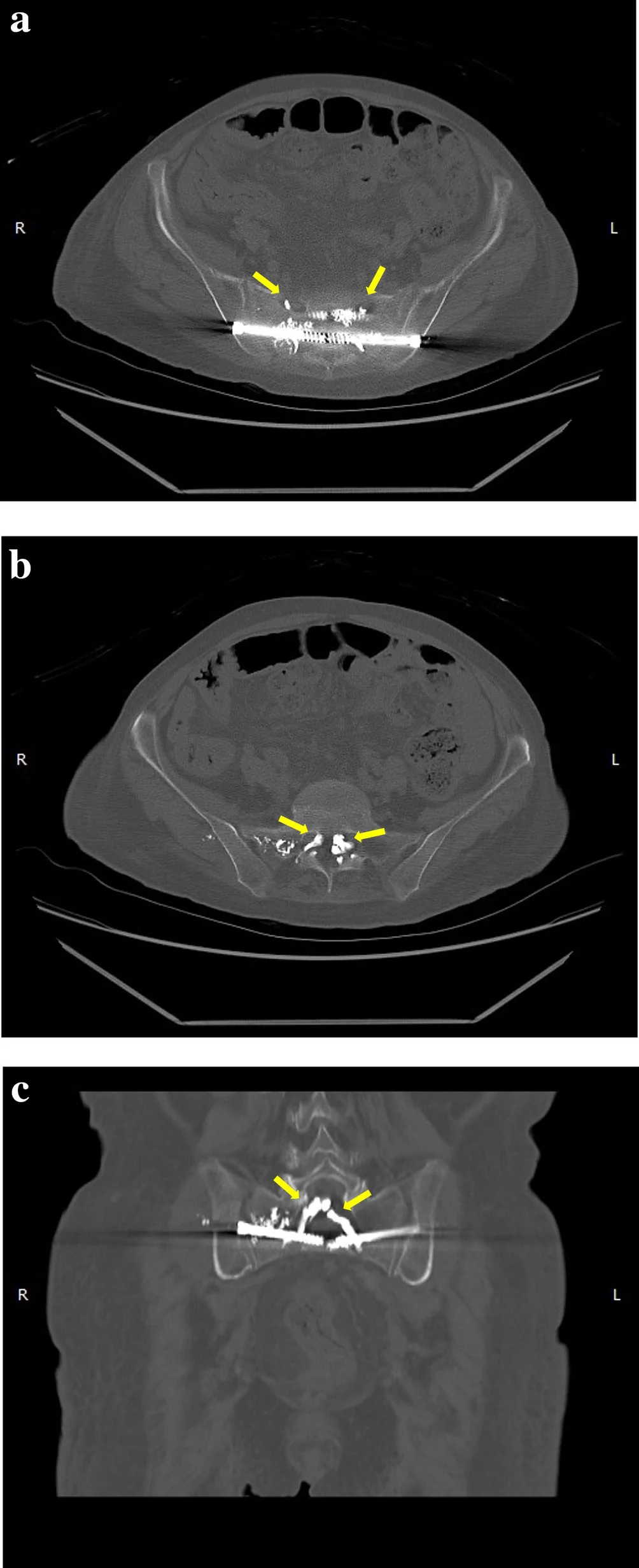
CT image showing unexpected cement extravasation into spinal canal (yellow arrows) after SI screw fixation and sacroplasty in axial view (a, b) and coronal view (c).

Nevertheless, one untoward complication occurred involving the unexpected extravasation of PMMA cement outside the fractured sacrum and into the spinal canal after sacroplasty. This patient was severely osteoporotic with a *T*‐score of −3.5 and was diagnosed with bilateral insufficiency sacral fractures. Bilateral S1 and S2 screws were inserted respectively followed by cement augmentation. Sacroplasty through S2 screws was first performed using low‐viscosity cement. However, extravasation was noted instantly on fluoroscopy after only 2 mL of cement was injected per screw. Sacroplasty procedures through S1 screws was therefore withheld. Postoperative CT was arranged at first clinic follow‐up demonstrating cement focal leakage into the spinal canal via screw holes (Figure 9). The screw positioning of this case was categorized into the “excellent” group. Given the concern of neurologic sequelae, this patient was followed‐up in a more frequent basis. Over a 2‐year surveillance period, the patient remained asymptomatic. No associated neurologic compromise, and no clinical evidence of venous emboli thrombosis was reported.

## 4 Discussion

Percutaneous fixation methods in treating sacral fractures are subjected to shorter operation time, less blood loss and reduced infection risks. However, it can be challenging as inaccurate placement of screws can possibly lead to severe complications. The incidence of screw malpositioning with fluoroscopic guidance has been reported from 3% to 32%, with nerve damage up to 8% [10, 11, 12, 13, 14]. Many methods to aid SI screw insertion such as O‐arm navigation, or CT‐guided fixation were proposed to increase accuracy and bring optimal safety to patients [15, 16]. While these advanced modalities offered precision, they came with high equipment costs and limited institutional availability. Our technique provides an alternative as conventional two‐dimensional fluoroscopy was enough to identify safe insertion corridors in SI screw placement.

### 4.1 Radiation Exposure and Screw Accuracy

Inspired by the pedicle‐guided screw placement technique in thoracolumbar spine under single fluoroscopy [17], we aimed at minimizing fluoroscopic shots and thus reducing radiation exposure to surgeons. Once the gantry was adjusted in correct position, screws could be safely placed by just utilizing the true sacral AP view and corresponding lateral view. Although it is hard to simply compare the number of fluoroscopic shots or time with previous studies or establish a control group, the radiation dosage of biplanar fluoroscopy alone is considerably lower than that of CT‐guided screw placement or conventional fluoroscopy which acquire additional inlet and outlet views during screw placement. The quality of screw positioning was examined according to Gras et al. Using our modified pedicle‐guided technique, screw excellence rate reached 97% and neither cortical breach of screw nor implant‐related nerve injuries was noted. No revision case was accounted. The accuracy rate in our study was comparable among the past literature documenting screw insertion under conventional fluoroscopic methods.

### 4.2 Learning Curve and Reproducibility

Surgeon's experience was thought to be a factor influencing the accuracy in screw placement [18]. Our 22 cases were collected from four different surgeons, with experience in spinal surgeries ranging from 2 to 25 years. Surprisingly of the two inadequately positioned screw, they came from the least and the most experienced surgeon, respectively. In terms of screw insertion time, the average time per screw was 14.9 min. Although the sample size was insufficient for analysis, we did not observe significant longer screw insertion time in young surgeons. Instead the discrepancy was noted when comparing placing the first screw and the rest. This was attributed to the fact that once the trajectory of first screw was confirmed, it was easy to modify the incision and pathway of the remaining screws. Preoperative CT or MRI is a helpful tool in evaluating the safe track and improving the entry point and the expected trajectory of SI screw. As for total operative time, longer duration was accounted when more screws were inserted or if screw fixation was combined with sacroplasty. We did not take additional procedures or concomitant treatment in count. In the most complex procedure which four screws were inserted with cement augmentation and sacroplasty, 70 min most was recorded by our technique. It was a comparable shorter length of time than that of previous literature. Notably, time for navigation setup regarding O‐arm or CT‐guided technique was spared as we used only two‐dimensional fluoroscopy. We also concluded that reducing attempts to obtain correct fluoroscopic shots by personnel under our method conduced to shorter operation time. Although we did not observe correlation with surgeon's experience, our surgical team was trained to insert thoracolumbar pedicle screws under single fluoroscopy [17]. Placing SI screw in pedicle‐guided technique may require a learning curve, especially when compared with navigation or CT‐guided fixation methods.

### 4.3 Surgical Considerations of Prone Positioning

Performing SI fixation in prone position has gained popularity nowadays. It gets rid of patient‐specific factors contributing to poor fluoroscopic visualization. In supine position, inadequate imaging may be caused by sacral dysmorphism, obesity and intestinal gas [7]. Obese patients were shown to have higher complication risks especially surgical site infection and poorer posttraumatic functional scores. Huge body mass could also hinder correct entry point during percutaneous screw placement leading to higher chance of root injury [19]. Of particular note, surgery was performed smoothly without prolonged operative time in an obese patient (body mass index 34.6). Prone positioning is also advantageous if open reduction is required in patients with large amount of abdominal fat. On the other hand, the dorsal‐to‐ventral trajectory of screw placement is more straightforward when the patient was in prone. Screw direction was from top to bottom and any adjustment made was more intuitive. Moreover, prone position is advisable for bilateral SI screw fixation as the procedure is less affected by body shape or operating room table. In supine position, the patient was placed to the table edge as possible in facilitating drill motion. However in situation when the individual was too skinny, surgical field in contralateral side became constrained. In addition, preparing the patient positioned prone offers a relatively more sterile field. Surgical draping is considered more complicated as posterior pelvic area needs to be elevated for better access in supine positioning.

Prone position is also superior when dealing with accompanied spinal injuries. In sacral insufficiency fractures or cases resulted from low‐energy fall, unstable thoracolumbar compression fractures often coexisted. It is also not rare that burst fractures and even chance fractures were seen in patients suffered from high‐energy trauma. In our study, nearly half the patients were presented with associated spinal injuries necessitating surgical attention. Patient being in prone allowed surgeons to perform concomitant spinal operations without repositioning, thus shortening total operation time and lowering risks from long anesthesia. Given that lateral‐compression type is the most common pelvic fracture pattern, prone positioning with a proper bolstering technique also helps self‐reduction as it allows the iliac bone to move outwards. However, prone positioning has its limitations. Supine position remains preferable in polytrauma patients with associated thoracoabdominal injuries and hemodynamic instability. The inability to simultaneously address the anterior pelvic ring makes prone position generally unsuitable for AP compression type or Tile B/C pelvic ring injuries necessitating anterior fixation. This also leads to the fact that our patient inclusion for traumatic sacral fractures had generally nondisplaced pelvic ring injury. Nevertheless, the modified pedicle‐guided technique can be extended to treat other posterior pelvic ring disruptions like sacral U‐type fractures or lumbosacral dissociation, provided that adequate bone corridors are present.

### 4.4 Clinical Outcomes and Functional Evaluation

The evaluation of SI screw fixation should not be limited to screw accuracy alone. In our study, clinical outcomes were assessed using the VAS and MPS. MPS measures functional components but lacks psychological and neurological evaluation which possesses prognostic value in long‐term recovery [20]. Nevertheless, this was not necessarily a limitation to our evaluation considering none of our patients develop neurological sequelae. They showed great pain improvement, mostly excellent functional recovery demonstrated by MPS and satisfactory final walking status. The outcomes were highly favorable, reflecting the predictable recovery potential of partially stable pelvic ring fractures without AP disruption seen in our cases as mentioned. On the contrary, when managing sacral fractures with unstable pelvic ring injuries, previous studies highlight the need for a broader outcome assessment. Incomplete reduction of pelvic ring can alter pelvic parameters such as pelvic tilt and sacral slope thus disrupting spinopelvic balance, leading to gait impairment and poor quality of life [21]. Additionally, Tile B and C injuries, particularly those associated with open‐book patterns and SI fractures were shown to a higher incidence of sexual dysfunction [22, 23, 24]. Therefore, future studies on prone SI screw fixation should include validated pelvic outcome scores, neurological examination, sexual function assessment when appropriate, gait or mobility outcomes, and longer‐term follow‐up.

### 4.5 Complication Management

No surgical site infection was found in our study. Despite quick recovery and mobilization with significant decrease in pain intensity over time in our patients, one event of cement leakage was observed. It is regarded as the most common complication in sacroplasty with an incidence of 3.3% [25]. In this case, the screws for sacroplasty were in appropriate trajectories. Extravasation of cement may be attributed to its low‐viscosity characteristic and the poor bone quality of the patient. Prone positioning may be advantageous in reducing the risk of cement leakage into spinal canal due to gravity effect. Preoperative imaging alerts surgeon to potential problems with respect to poor bone quality. Additionally, high‐viscosity cement is preferable in osteoporotic patients.

### 4.6 Study Limitations

The retrospective nature with the absence of control group and a small sample size is considered the major limitations of our study. Various degrees of injuries and different fracture patterns may have affected functional outcomes. We therefore focused mainly on examining the accuracy in screw placement which our technique brought to minimize random variations. We suggest more cases for future studies and the procedures being an alternative approach in treating sacral fractures.

## 5 Conclusion

Our proposed technique proved to be a feasible approach for SI screw fixation, demonstrating satisfactory preliminary radiographic and clinical outcomes in a selected cohort. It aims at decreasing operative time and minimizing radiation exposure to surgeons, at the same time less facility‐demanding.

## Author Contributions

**Ben‐Mao Liu:** data curation, writing – review and editing, writing – original draft. **Yu‐Hsin Liu:** methodology, validation. **Ting‐Shuo Hsu:** methodology, validation. **Chih‐Yu Chen:** methodology, validation. **Chang‐Jung Chiang:** conceptualization, methodology, validation. **Yueh‐Ying Hsieh:** conceptualization, methodology, validation, supervision.

## Funding

The authors have nothing to report.

## Conflicts of Interest

The authors declare no conflicts of interest.

## Data Availability

The data that support the findings of this study are available from the corresponding author upon reasonable request.
